# Supplementary material for: Efficient GHz electro-optical modulation with a nonlocal lithium niobate metasurface in the linear and nonlinear regime
Source: Nat Commun. 2025 Jul 30;16:7000. doi: 10.1038/s41467-025-62072-7 (PMC12310964; doi:10.1038/s41467-025-62072-7)
Supplement: Supplementary file 1 — Supplementary Information [file 41467_2025_62072_MOESM1_ESM.pdf]

Supplementary Information

Agostino Di Francescantonio[1], Alessandra Sabatti[2], Helena Weigand[2], Elise Bailly-Rioufreyt[2], Maria Antonietta Vincenti[3], Luca Carletti[3], Jost Kellner[2], Attilio Zilli[1], Marco Finazzi[1], Michele Celebrano[1*], and Rachel Grange[2*]

[1] Politecnico di Milano, Physics Department, Milano, Italy
[2] ETH Zurich, Department of Physics, Institute for Quantum Electronics, Optical Nanomaterial Group, 8093 Zurich, Switzerland
[3] Università di Brescia, Department of Information Engineering, Brescia, Italy

*Correspondence to: michele.celebrano@polimi.it; grange@phys.ethz.ch

S1. Experimental Setup

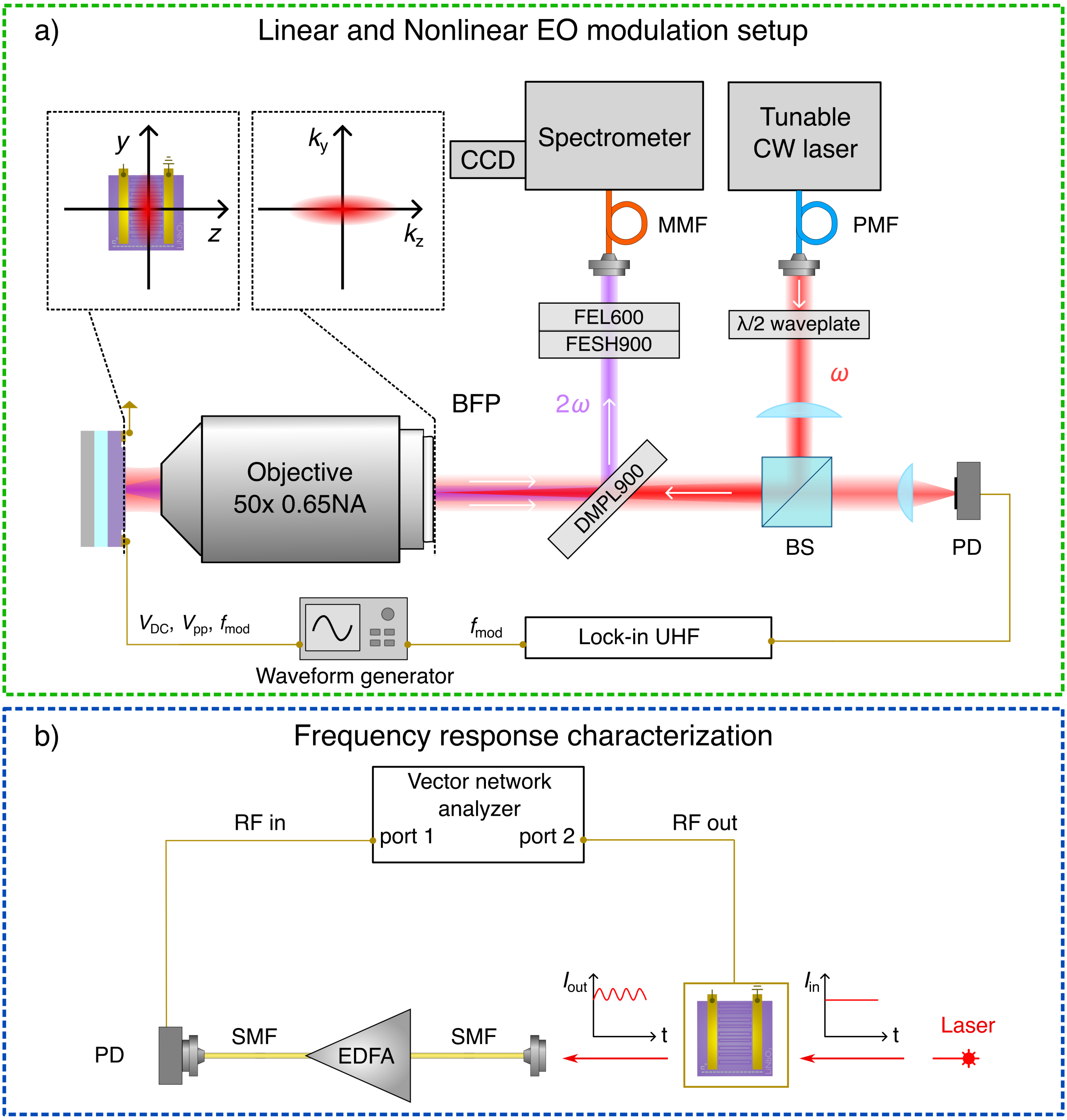

Figure S1 Schematic of the experimental setups. **a)** Linear and nonlinear characterization of the electro-optic (EO) modulation. Acronyms: CW= continuous wave, MMF = multimode fiber, PMF = polarization-maintaining fiber, BS = beam splitter, BFP = back-focal plane, DMLP = long pass dichroic mirror, UHF = ultra-high frequency. **b)** Optoelectronic characterization by means of a vector-network analyzer (VNA). Acronyms: RF = radiofrequency, SMF = single-mode fiber, EDFA = erbium-doped fiber amplifier.

S2. Numerical simulations with COMSOL

The metasurfaces linear spectra, the refractive index change induced by Pockels effect and the second-harmonic generation (SHG) presented in this article have all been simulated by Finite-Element Method (FEM) using COMSOL Multiphysics® 6.2. The translational invariance along z allows us to implement a 2D unit cell (see Figure 2b and Figure S4, S5 of the Supplementary Information), reducing the computational workload. We set a periodicity $p = 800$ nm for the supercell, which includes two asymmetric nanowires (150 nm high on top of 450 nm $LiNbO_3$ film). The $LiNbO_3$ ordinary and extraordinary index dispersion are described via Sellmeier equation [1]. We take into account the tilted sidewalls resulting from fabrication with an angle of $\theta = 65°$. The LiNnO3 grating is placed on top of a SiO_2 layer ($h_{sub} = 2$ µm, $n_2 = 1.45$) and Si (semi-infinite, $n_3 = 3.69$ at 775 nm [2] and $n_3 = 3.48$ at 1550 nm [3]). Floquet periodic boundary conditions are defined at the unit cell lateral boundaries to mimic an infinitely extended periodic structure. The upper semi-infinite space is made by air ($n_1 = 1$). We implement a three-steps simulation:

1. The change in the refractive index is calculated within a static field interface, available in the AC/DC module. For this step we define a 3D unit cell (see Figure S3) to calculate the static electric field $\mathbf{E}_{EO}$ subtended by in-plane electrodes spaced by 14 µm. The static voltage V_0 is applied to a 300 nm – height Electric Potential port (the one on the other side of the electrodes is set to ground). Thanks to the uniformity of $\mathbf{E}_{EO}$ in the $LiNbO_3$ film (see Figure S3), only the solution for the z component $E_{EO,z}$ is used to calculate the modifications of the refractive index induced by the Pockels effect [4]:

$$\begin{cases} n_x(\lambda,x,y,z) \approx n_0(\lambda) - \dfrac{1}{2} r_{13}(\lambda) n_0^3(\lambda) E_{\mathrm{EO},z}(x,y,z), \\ n_y(\lambda,x,y,z) \approx n_0(\lambda) - \dfrac{1}{2} r_{13}(\lambda) n_0^3(\lambda) E_{\mathrm{EO},z}(x,y,z), \\ n_z(\lambda,x,y,z) \approx n_e(\lambda) - \dfrac{1}{2} r_{33}(\lambda) n_e^3(\lambda) E_{\mathrm{EO},z}(x,y,z), \end{cases} \tag{1}$$

   with $r_{13} = 10.3$ pm/V and $r_{33} = 34.1$ pm/V [5] for $\lambda = 1.32$ µm, assumed to be the same at 1550 nm [6], and $r_{13} = 11$ pm/V and $r_{33} = 36.7$ pm/V for λ in the visible range [5].
2. The linear reflectance is calculated with a scattered-field formulation of the frequency domain interface of the wave optics module. We exploit the invariance along z and model the 2D unit cell as described above. The top port shown in Figure S4 of the models a plane-wave excitation, impinging at normal incidence and polarized along the z axis for TE simulations (i.e., orthogonal to the simulation plane). Top and bottom ports work also to absorb the reflected and transmitted light, thus mimicking infinite Si and air layers in the –x and +x directions, respectively. The Pockels effect is included by performing a static field simulation (step 1) and extracting the averaged field E_{EO} in the $LiNbO_3$ layer. The electric field is indeed homogeneous in any xy cross section with almost no variation along z far away from the electrodes (see Figure S3b,c). Therefore, we know the $LiNbO_3$ refractive index change for any given input voltages V_0 with Equation 1.
3. SHG is calculated in a full-field, frequency domain formulation at twice the fundamental frequency. Under the hypothesis of undepleted pump, we use the solution of the optical electric field obtained from step 2 as fundamental field $E_{\mathrm{i}}(\omega)$ for the computation of the nonlinear current density $\mathbf{J}(2\omega) = -2i\omega\mathbf{P}^{(2)}(2\omega)$. The nonlinear polarization $\mathbf{P}^{(2)}(2\omega)$ takes the form:

$$\begin{cases}\begin{bmatrix}P_x^{(2)}(2\omega)\\ P_y^{(2)}(2\omega)\\ P_z^{(2)}(2\omega)\end{bmatrix} = 2\epsilon_0\begin{bmatrix}2d_{31}E_x(\omega)E_z(\omega)-2d_{22}E_x(\omega)E_y(\omega)\\ -d_{22}E_x(\omega)^2+d_{22}E_y(\omega)^2+2d_{31}E_y(\omega)E_z(\omega)\\ d_{31}E_x(\omega)^2+d_{31}E_y(\omega)^2+d_{33}E_z(\omega)^2\end{bmatrix}\end{cases}, \quad (2)$$

which simplifies into $P_z(2\omega) = 2\epsilon_0 d_{33}E_z(\omega)^2$. To calculate the power radiated in the upper space we compute the Poynting vector flux $\int \mathbf{\Pi}\cdot \mathrm{d}\mathbf{l}$, through the top-port boundary, where $\mathbf{\Pi}$ is the time-averaged Poynting vector and $\mathrm{d}\mathbf{l}$ the outgoing vector normal to the top port (see Figure S4). Perfectly matched layers have also been added after the linear ports for the SH computation to prevent any back reflection into the simulation volume. The Pockels effect has been considered in both steps of the second-harmonic computation by modifying the refractive index of the $LiNbO_3$ according to Sellmeier equations. In the case of the SH simulations, we took the values of the electro-optic coefficients measured at 632.8 nm [5]. As for the fundamental step, we used the average value of the static field in $LiNbO_3$ to compute the change of the refractive index. Also, the second order nonlinear coefficients d_{ij} are unknown at 1.5 μm. We thus used values measured for the fundamental wavelength at 1.313 μm (d_{31} = −4.3 pm/V, d_{33} = −27 pm/V)[7], while d_{22} = 2.1 pm/V [8].

Material properties

Lithium niobate ($LiNbO_3$) is a birefringent material where the dispersion of the principal extraordinary refractive index n_e and the principal ordinary refractive index n_o is described via the Sellmeier equations given in Equation 1 and 2. The Sellmeier coefficients are taken from [1]. The extraordinary index is associated with the *z* axis in the COMSOL implementation.

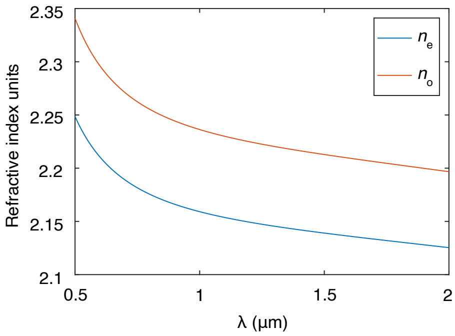

Figure S2 Extraordinary and ordinary refractive indices (n_e and n_o respectively) as a function of the wavelength. The analytical expressions of ne and no are given by Equation 1 and 2.

$$n_e(\lambda) = \sqrt{1+\frac{2.9804\,\lambda^2}{\lambda^2-0.02047}+\frac{0.5981\,\lambda^2}{\lambda^2-0.0666}+\frac{8.9543\,\lambda^2}{\lambda^2-416.08}}, \quad (1)$$

$$n_o(\lambda) = \sqrt{1+\frac{2.6734\,\lambda^2}{\lambda^2-0.01764}+\frac{1.2290\,\lambda^2}{\lambda^2-0.05914}+\frac{12.614\,\lambda^2}{\lambda^2-474.60}}, \quad (2)$$

where λ is given in μm. The dispersion of n_e and n_o as a function of the wavelength is plotted in Figure S2.

For silicon, we use the refractive index from [3] for the linear case, and a constant value from [2] for the nonlinear simulations (n_{Si} (λ = 775 nm) = 3.69).

For silicon-oxide (SiO_2) we used the refractive index n_{SiO2} = 1.45 from [9] for both linear and nonlinear simulations

The dielectric constants values used for the numerical computation of the static field are listed below:

- $LiNbO_3$: the unclamped values have been used, ϵ_{11}^{T} = 84.2, ϵ_{33}^{T} = 29.2 [10],
- Si: ϵ = 11.7 [11],
- SiO_2: ϵ = 3.7 [12]

The nonlinear coefficients used in the linear and nonlinear simulations are summarized in Table 1.

	λ = 1500 nm	λ = 775 nm
r_{13} (pm/V)	10.3 ($\lambda = 1320$ nm) [5]	11 ($\lambda = 632.8$ nm) [5]
r_{33} (pm/V)	34.1 ($\lambda = 1320$ nm) [5]	36.7 ($\lambda = 1320$ nm) [5]
d_{13} (pm/V)	-	−4.3 ($\lambda_{FW} = 1320$ nm) [7]
d_{22} (pm/V)	-	2.1 ($\lambda_{FW} = 1152$ nm) [8]
d_{33} (pm/V)	-	−27 ($\lambda_{FW} = 1320$ nm) [7]

Table S1 Summary of the nonlinear coefficients used in the numerical simulations (for Pockels effect and SHG), with the wavelength at which they have been measured in literature. In the case of the SHG, the wavelength corresponds to the fundamental one

Simulation geometry

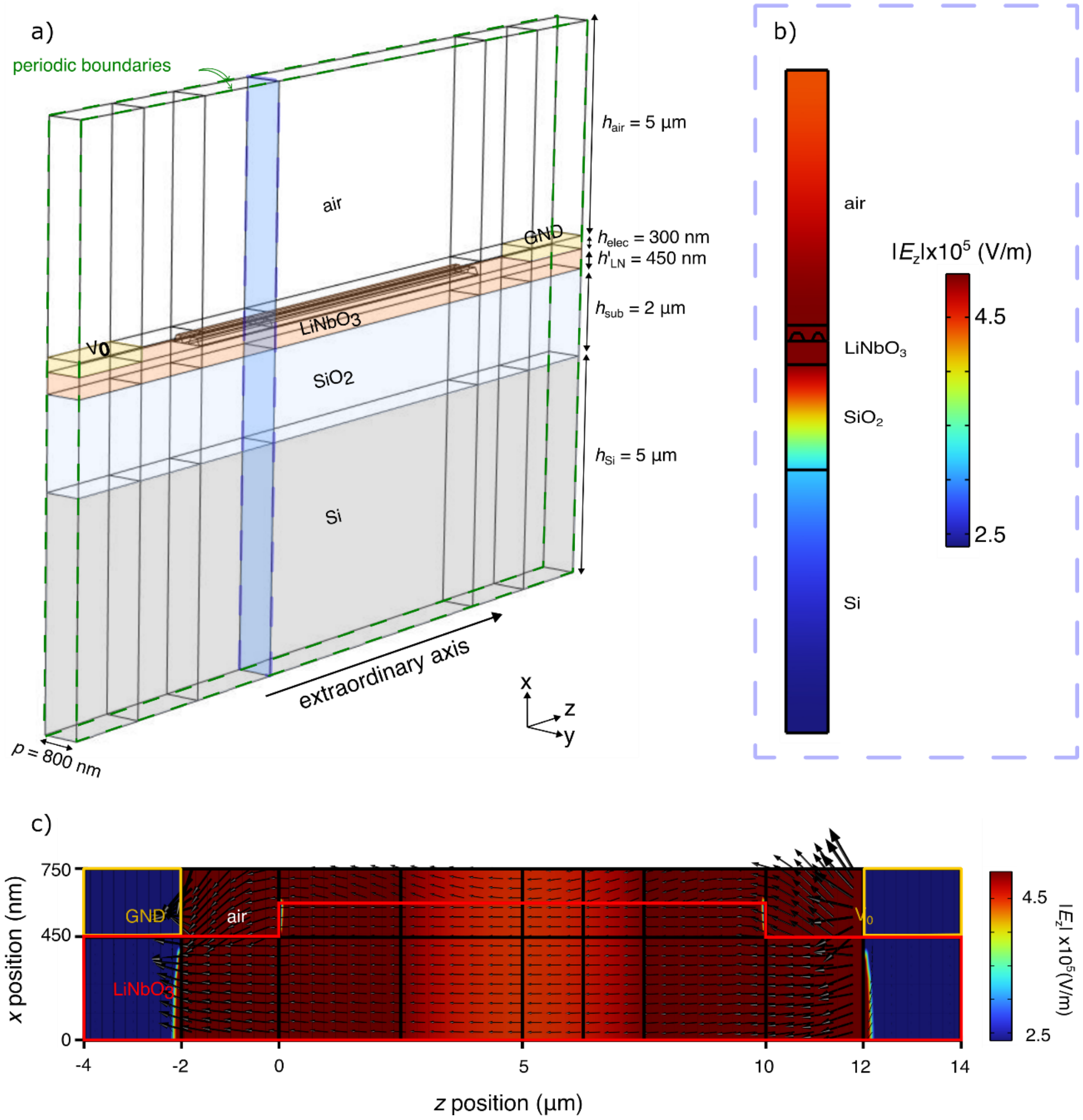

Figure S3 COMSOL cell for the calculation of the static electric field distribution. **a)** Geometry of the simulated unit cell. The voltage V_0 is applied at one electrode, while the other one is grounded (GND). Periodic conditions are applied to the surfaces perpendicular to the *z*-axis. **b)** Norm of the amplitude of the static field component E_z, plotted for a specific *xy* transverse section (highlighted in blue in a), at z = 2.5 µm, for $V_0 = 9$ V, showing a homogeneous field distribution inside the Lithium Niobate ($LiNbO_3$) structure. **c)** Normalized amplitude of the component E_z of the static electric field in a *xz* transverse section (dimensions are not on same scale), plotted in the center of the left bar, at $y = -p/4$. The arrowfield shows the electric field direction, being mostly along *z* in the $LiNbO_3$

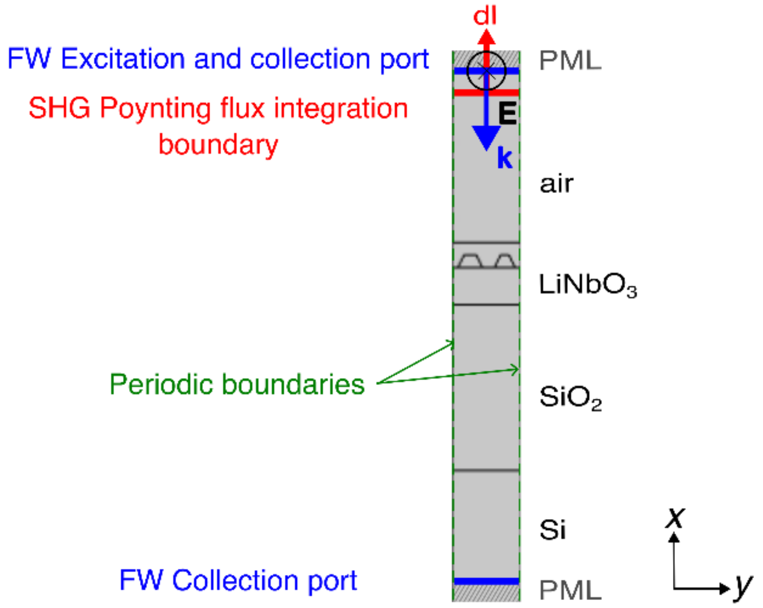

Figure S4 2D COMSOL cell for the calculation of the optical field distribution and reflectance and transmittance (dimensions along the *x* and *y* axes are not on the same scale). PML indicates a perfectly matched boundary condition. The fundamental field **E** impinges at normal incidence (**k** wavevector parallel to the *x* axis) from the top input port (blue boundary on the top) and polarized along *z* for TE simulations. For the second harmonic simulations, a perfectly matched layer is added, and the Poynting vector flux $\int \mathbf{\Pi} \cdot \mathbf{dl}$, where $\mathbf{\Pi}$ is the time-averaged Poynting vector and $\mathbf{dl}$ the outgoing vector normal to the top port (red boundary), is evaluated.

S3. Linear and electro-optic characterization of the different designs

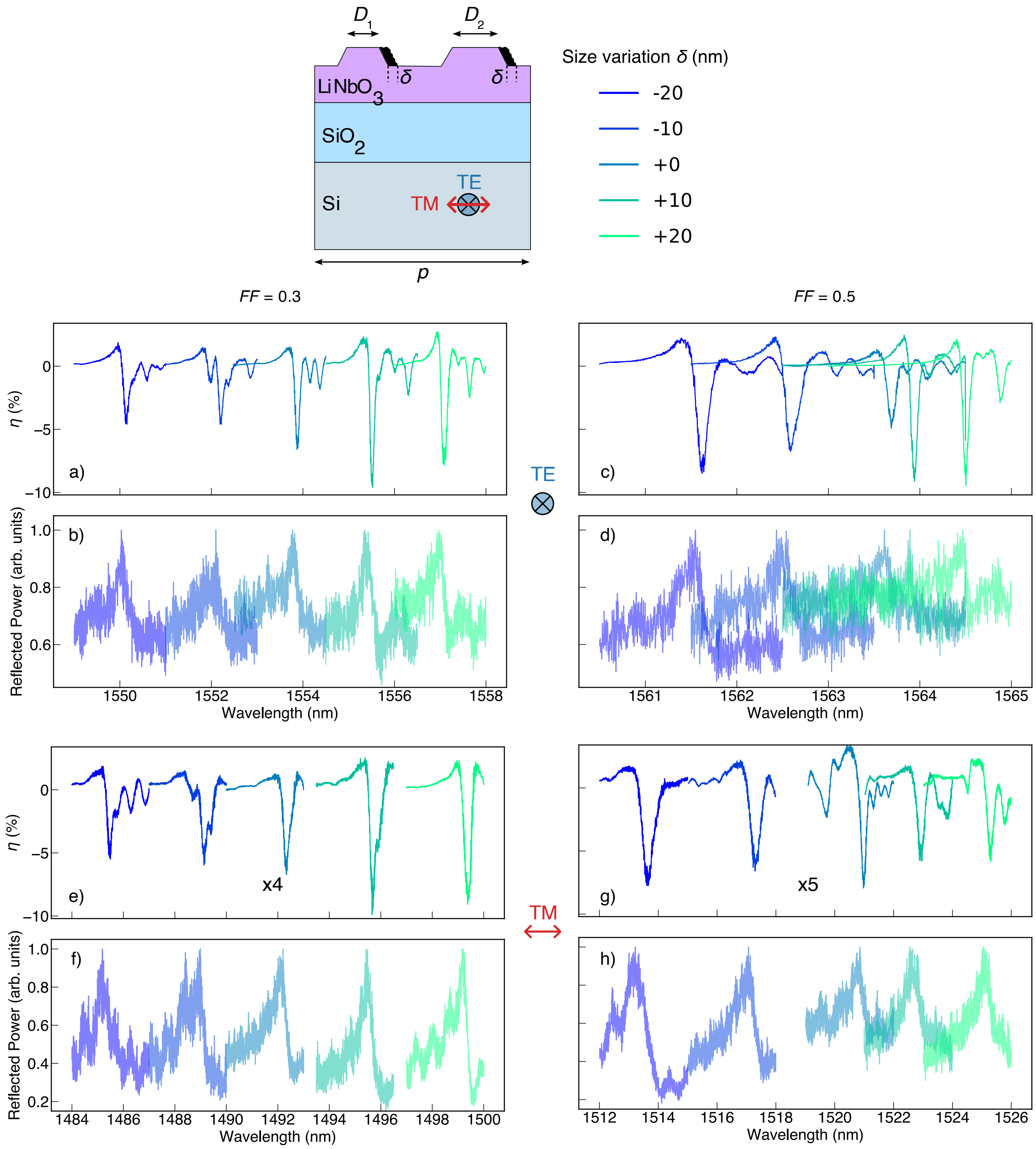

Figure S5 Measured reflected power and electro-optic response depending on geometry and polarization. Within each macro-design (identified by the fill factor *FF*), the size of D_1 and D_2 is changed by an amount δ (see sample transverse section on the top). **a),c),e)** and **g)** report the modulation efficiency $\eta = P_\omega(f = f_r)/P_\omega(f = 0)$; **b),d),f)** and **h)** report the corresponding reflectance spectra. The data in the top (bottom) four panels are collected with TE (TM) pump polarization. The sample is gated by a sinusoidal drive with peak-to-peak amplitude V_{pp} = 10 V and frequency f_{mod} = 100 kHz.

S4 Second-harmonic spectrum

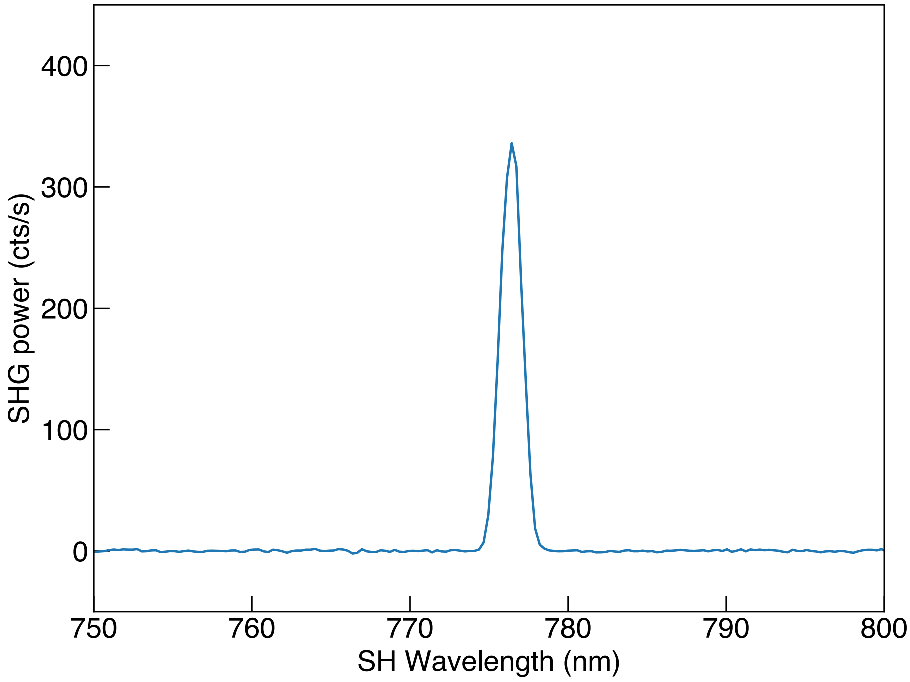

Figure S6 SHG emission spectrum from the sample with *FF* = 0.3 and α = 0.19, excited at the fundamental wavelength λ = 1553.99 nm with a CW optical intensity of 13 kW/cm^2. The spectrum FWHM is limited by the spectrometer resolution (2 nm).

S5 Second-harmonic simulations

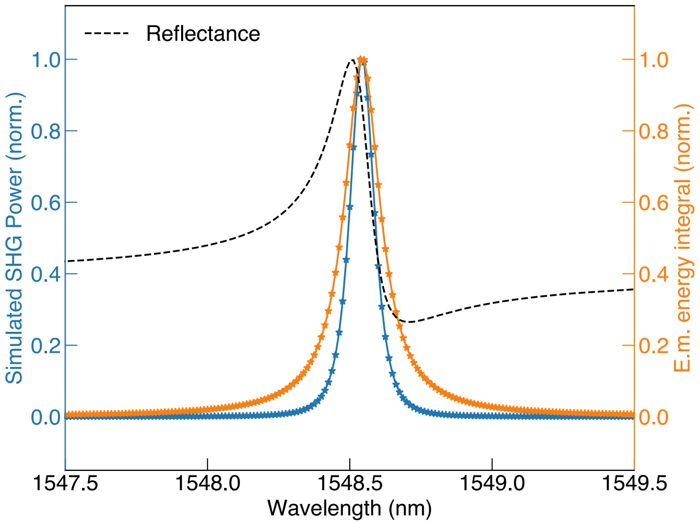

Figure S7 Simulated second-harmonic excitation spectrum. In blue, simulated second-harmonic power irradiated by the metasurface with *FF* = 0.3 and α = 0.19 in the upper part of space. Each point is obtained by integrating the SHG Poynting vector over the upper boundary of the simulation cell. The orange data are obtained by integrating the electromagnetic energy density $u_{EM} = \frac{\epsilon_0}{4}[n_o^2|\boldsymbol{E}_o^2| + n_e^2|\boldsymbol{E}_e^2|]$, over the LN volume where $n_{o,e}$ are the ordinary and extraordinary refractive indices. The dashed line is the calculated reflectance spectrum.

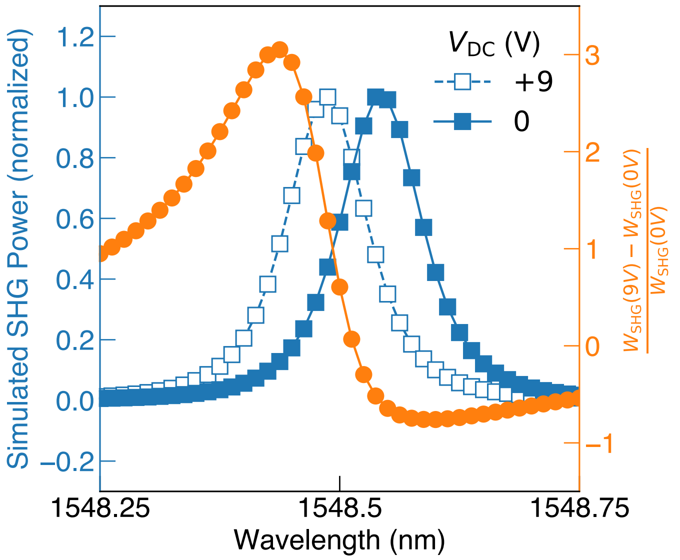

Figure S8 Simulated second-harmonic modulation amplitude. In blue, second-harmonic excitation spectra calculated for the geometry *FF* = 0.3 and α = 0.19, V_{DC} = ± 9 V. The orange data indicate the SHG modulation efficiency, calculated from the blue curves with the same definition of the article $(P_{SHG}(9\,V) - P_{SHG}(0\,V))\,/\,P_{SHG}(0\,V)$.

S6. SHG from the sample with *FF* = 0.5, α = 0.2 and from unpatterned LN

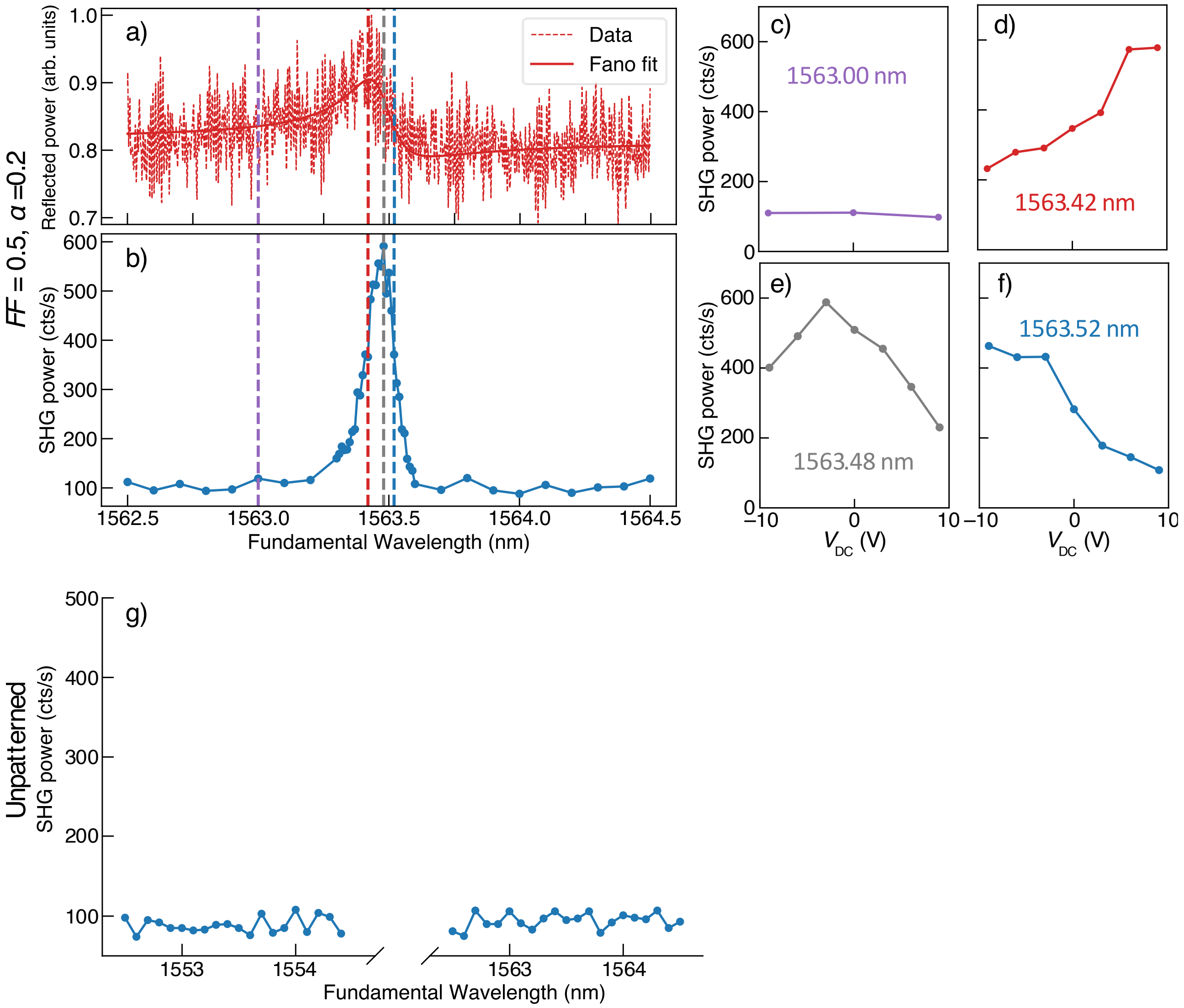

Figure S9 a) Spectrum of the reflected signal from the sample with *FF* = 0.5 and α = 0.19. **b)** Second-harmonic power as a function of the fundamental wavelength. The fundamental optical intensity on the sample is estimated to be 12 kW/cm^2. **c–f)** SHG power variation by exciting with four wavelengths (indicated by vertical, dashed lines in panels a,b) depending on the applied DC voltage V_{DC}. **g)** SHG power detected from the unpatterned LN on the same sample stack (h'_{LN} = 450 nm) in the two pump wavelength ranges of the samples investigated in the main (see Figure 4) and in the Supplementary Information.

References

[1] D. E. Zelmon, D. L. Small, and D. Jundt, “Infrared corrected Sellmeier coefficients for congruently grown lithium niobate and 5 mol% magnesium oxide –doped lithium niobate,” *Journal of the Optical Society of America B*, vol. 14, no. 12, p. 3319, Dec. 1997, doi: 10.1364/JOSAB.14.003319.

[2] C. Schinke *et al.*, “Uncertainty analysis for the coefficient of band-to-band absorption of crystalline silicon,” *AIP Adv*, vol. 5, no. 6, Jun. 2015, doi: 10.1063/1.4923379.

[3] C. D. Salzberg and J. J. Villa, “Infrared Refractive Indexes of Silicon Germanium and Modified Selenium Glass*,” *J Opt Soc Am*, vol. 47, no. 3, p. 244, Mar. 1957, doi: 10.1364/JOSA.47.000244.

[4] R. W. Boyd, “Chapter 11 - The Electrooptic and Photorefractive Effects,” in *Nonlinear Optics (Third Edition)*, Third Edition., R. W. Boyd, Ed., Burlington: Academic Press, 2008, pp. 511–541. doi: https://doi.org/10.1016/B978-0-12-369470-6.00011-3.

[5] R. J. Holmes, Y. S. Kim, C. D. Brandle, and D. M. Smyth, “Evaluation of crystals of LiNbo3 doped with MgO or TiO2 for electrooptic devices,” *Ferroelectrics*, vol. 51, no. 1, pp. 41–45, Nov. 1983, doi: 10.1080/00150198308009051.

[6] J. L. Casson *et al.*, “Electro-optic coefficients of lithium tantalate at near-infrared wavelengths,” *Journal of the Optical Society of America B*, vol. 21, no. 11, p. 1948, Nov. 2004, doi: 10.1364/JOSAB.21.001948.

[7] I. Shoji, T. Kondo, A. Kitamoto, M. Shirane, and R. Ito, “Absolute scale of second-order nonlinear-optical coefficients,” *Journal of the Optical Society of America B*, vol. 14, no. 9, p. 2268, Sep. 1997, doi: 10.1364/JOSAB.14.002268.

[8] M. J. Weber, *Handbook of Optical Materials*. CRC Press, 2018. doi: 10.1201/9781315219615.

[9] I. H. Malitson, “Interspecimen Comparison of the Refractive Index of Fused Silica*,†,” *J Opt Soc Am*, vol. 55, no. 10, p. 1205, Oct. 1965, doi: 10.1364/JOSA.55.001205.

[10] I. Tomeno and S. Matsumura, “Elastic and Dielectric Properties of $LiNbO_3$,” *J Physical Soc Japan*, vol. 56, no. 1, pp. 163–177, Jan. 1987, doi: 10.1143/JPSJ.56.163.

[11] W. C. Dunlap and R. L. Watters, “Direct Measurement of the Dielectric Constants of Silicon and Germanium,” *Physical Review*, vol. 92, no. 6, pp. 1396–1397, Dec. 1953, doi: 10.1103/PhysRev.92.1396.

[12] P. P. Budnikov and Y. E. Pivinskii, “QUARTZ CERAMICS,” *Russian Chemical Reviews*, vol. 36, no. 3, pp. 210–227, Mar. 1967, doi: 10.1070/RC1967v036n03ABEH001599.
